# Intervention research on cultivating psychological resilience in adolescent athletes: an empirical analysis based on mindfulness training

**DOI:** 10.3389/fpsyg.2025.1589786

**Published:** 2025-12-04

**Authors:** Shanshan Wang, Jingwu Liu

**Affiliations:** Department of Physical Education, Changzhi University, Changzhi, Shanxi, China

**Keywords:** mindfulness training, psychological resilience, adolescent athletes, sports psychology, mental health intervention

## Abstract

**Introduction:**

Psychological resilience is crucial for adolescent athletes facing competitive pressures, yet effective interventions remain understudied. This study examined the associations between mindfulness training and psychological resilience development in adolescent athletes.

**Methods:**

A randomized controlled trial was conducted with 60 adolescent athletes (aged 14-18 years) from a provincial sports school in China. Participants were randomly assigned to an experimental group (*n* = 30) receiving an 8-week mindfulness training program (90 minutes weekly) or a control group (*n* = 30) maintaining regular training routines. Psychological resilience was assessed using the Connor-Davidson Resilience Scale at baseline, post-intervention, and 3-month follow-up. Potential moderating effects of gender and training experience were analyzed.

**Results:**

The experimental group demonstrated significant improvements in total resilience scores from pre-intervention (*M* = 62.47, SD = 10.21) to post-intervention (*M* = 75.83, SD = 9.56, *p* < 0.001, *d* = 1.35) and follow-up (*M* = 77.90, SD = 9.14, *p* < 0.001, *d* = 1.58), while the control group showed no significant changes. Improvements were observed across all resilience dimensions: personal competence, trust in instincts, positive acceptance of change, and control (all *p* < 0.001). Neither gender nor training experience significantly moderated intervention effects.

**Discussion:**

These findings provide empirical support for associations between mindfulness training and enhanced psychological resilience in adolescent athletes, with sustained effects at 3-month follow-up. The intervention’s effectiveness across gender and experience levels suggests broad applicability for mental health promotion in youth sports programs. Future research should examine causal mechanisms and long-term sustainability with larger, more diverse samples.

## 1 Introduction

### 1.1 Research background

Psychological resilience, defined as the ability to cope with and adapt positively to stress and adversity, is a crucial factor in the growth and development of adolescent athletes (Balcombe et al., 2022). Numerous studies have shown that higher levels of psychological resilience are associated with better mental health, enhanced athletic performance, and lower risk of burnout in young athletes (Galli and Vealey, 2008; Harman et al., 2022). As the demands and pressures faced by adolescent athletes continue to rise, there is a growing need for effective interventions to cultivate their psychological resilience (Nuetzel et al., 2023).

Mindfulness training, which involves focusing one’s attention on the present moment without judgment, has emerged as a promising approach for enhancing resilience (Brenner, 2024). A growing body of research suggests that mindfulness-based interventions can reduce stress, improve emotional regulation, and promote well-being in various populations (Wang et al., 2023; Myall et al., 2023). However, the application of mindfulness training in the context of adolescent athletes’ resilience development remains understudied (Su et al., 2024).

### 1.2 Research significance

Investigating the effects of mindfulness training on psychological resilience in adolescent athletes has important theoretical and practical implications. From a theoretical perspective, this study can contribute to the understanding of the mechanisms underlying resilience development and the potential role of mindfulness in this process (Fletcher and Sarkar, 2012). The findings may also shed light on the generalizability of mindfulness interventions across different populations and contexts (Den Hartigh et al., 2022).

Practically, identifying effective strategies for enhancing resilience in young athletes is crucial for promoting their mental health, holistic development and long-term well-being (Hussain et al., 2023). Mindfulness-based interventions show promise for integration into youth development programs and mental health promotion initiatives, providing adolescents with valuable psychological tools to navigate developmental challenges and stress management both in sports and daily life (Roth et al., 2024). This approach emphasizes preventive mental health care, particularly important in competitive sports environments where young athletes face comprehensive demands that may impact their psychological well-being.

### 1.3 Research objectives

The primary objective of this study is to explore the associations between mindfulness training intervention and psychological resilience development among adolescent athletes. This research aims to contribute to mental health promotion and well-being enhancement in youth sports contexts. Specifically, we aim to:

1. Evaluate the relationship between an 8-week mindfulness training program and various dimensions of psychological resilience in adolescent athletes (Chen et al., 2024).
2. Analyze potential moderating factors including gender and training experience on the associations between mindfulness intervention and resilience outcomes (Li et al., 2023).
3. Examine the sustainability of mindfulness training-related changes in resilience over a 3-month follow-up period (Gupta and McCarthy, 2022).

### 1.4 Research innovation

This study advances the existing literature in several ways. First, it represents one of the few empirical investigations into the application of mindfulness training for resilience development specifically among adolescent athletes. Second, by employing a rigorous experimental design with a control group and multiple assessment points, we can examine associations and relationships related to the intervention. Finally, by exploring potential mediators and moderators, this study can provide a more nuanced understanding of how and for whom mindfulness training may be most beneficial in the context of youth sports.

## 2 Literature review and theoretical foundations

### 2.1 Conceptualization and research status of psychological resilience

Psychological resilience has been a subject of growing interest in recent years, with researchers seeking to understand its nature, antecedents, and consequences (Fletcher and Sarkar, 2013). The concept of resilience originated from developmental psychology, referring to the capacity of individuals to maintain or regain mental health despite experiencing adversity (Rutter, 2006). In the context of sports, psychological resilience is often defined as the ability to positively adapt to stress, setbacks, and challenges encountered in athletic pursuits (Sarkar and Fletcher, 2014).

Research has identified several key characteristics of psychologically resilient athletes. These include high self-efficacy, positive coping strategies, emotional intelligence, and strong social support networks (Galli and Gonzalez, 2015; Hosseini and Besharat, 2010). Resilient athletes are able to view challenges as opportunities for growth, maintain focus and motivation in the face of obstacles, and bounce back quickly from failures or setbacks (Hill et al., 2018).

The development of psychological resilience in athletes is influenced by a complex interplay of individual, social, and environmental factors (Kalkhoven et al., 2020). Individual factors such as personality traits, cognitive appraisal styles, and mental skills have been shown to contribute to resilience (Collins and MacNamara, 2012). Social support from coaches, teammates, and family members also plays a crucial role in fostering resilience (Hardy et al., 2010). Environmental factors, including the motivational climate and organizational culture within sports teams and institutions, can also impact athletes’ resilience (Seery, 2011).

Measuring psychological resilience in sports contexts has been approached from various angles. Some studies have adapted general resilience scales, such as the Connor-Davidson Resilience Scale (CD-RISC), for use with athlete populations (Connor and Davidson, 2003). Others have developed sport-specific measures, like the Athletic Coping Skills Inventory-28 (ACSI-28), which assesses psychological skills and coping strategies associated with resilience (Smith et al., 1995). Qualitative approaches, such as interviews and case studies, have also been used to gain a deeper understanding of athletes’ resilience experiences (Gucciardi et al., 2011).

Despite the growing body of research on psychological resilience in sports, there remain gaps in understanding how resilience can be effectively cultivated, particularly among adolescent athletes (Wagstaff et al., 2018). While some studies have examined the effects of mental skills training and positive youth development programs on resilience, the potential of mindfulness-based interventions in this context has been largely unexplored (Gonzalez et al., 2016). This highlights the need for further empirical investigation into the application of mindfulness training for enhancing resilience in young athletes.

### 2.2 Application of mindfulness training in sports psychology research

Mindfulness, a concept rooted in Buddhist philosophy and meditation practices, has gained increasing attention in sports psychology over the past two decades (Baltzell, 2016). Mindfulness is commonly defined as the state of being aware and attentive to present moment experiences, without judgment or reactivity (Brown and Ryan, 2003). In the context of sports, mindfulness training aims to cultivate athletes’ ability to maintain present-moment focus, accept and regulate emotions, and enhance performance under pressure (Kaufman et al., 2018).

Various mindfulness-based interventions have been adapted and applied in sports settings. One widely used approach is Mindfulness-Acceptance-Commitment (MAC), which combines mindfulness techniques with acceptance and commitment therapy principles to improve athletes’ psychological flexibility and performance (Gardner and Moore, 2007). Another popular program is Mindful Sport Performance Enhancement (MSPE), which integrates mindfulness meditation with traditional mental skills training (Kaufman et al., 2009). These interventions typically involve a combination of formal meditation practices (e.g., breath awareness, body scan) and informal mindfulness exercises (e.g., mindful stretching, mindfulness during sport-specific movements) (Birrer et al., 2012).

Empirical studies have demonstrated the positive effects of mindfulness training on various aspects of athletes’ psychological functioning and performance. A systematic review by Noetel et al. (2019) found that mindfulness interventions significantly reduced stress, anxiety, and burnout among athletes, while improving emotional regulation, attentional control, and flow experiences. Specific to performance outcomes, a meta-analysis by Bühlmayer et al. (2017) reported small to moderate positive effects of mindfulness on athletes’ performance across a range of sports.

In addition to these general benefits, mindfulness training has been shown to enhance specific mental skills relevant to sports performance. For example, a study by Josefsson et al. (2017) found that a mindfulness intervention improved elite golfers’ ability to cope with performance anxiety and maintain focus during competition. Similarly, a case study by Bernier et al. (2009) demonstrated how mindfulness practice helped an Olympic swimmer develop greater awareness, acceptance, and trust in his body during high-pressure races.

Despite the growing evidence supporting the efficacy of mindfulness interventions in sports, there remain limitations and gaps in the current research. Many studies have focused on adult and elite athletes, with less attention given to the potential benefits for adolescent and developing athletes (Reardon et al., 2019). Additionally, the mechanisms through which mindfulness training enhances performance and well-being in sports contexts are not fully understood, highlighting the need for further research into potential mediators and moderators (Josefsson et al., 2024).

The application of mindfulness training to specific areas of sports psychology, such as resilience development, is an emerging area of inquiry. While some studies have suggested that mindfulness may contribute to resilience by promoting adaptive coping, emotion regulation, and cognitive flexibility (Lazarus and Folkman, 1984; Fredrickson, 2004; Ryan and Deci, 2020), empirical research directly examining the effects of mindfulness interventions on athletes’ resilience is limited. This underscores the importance of the current study in advancing our understanding of how mindfulness training can be harnessed to cultivate psychological resilience in adolescent athletes.

### 2.3 Theoretical foundations

The current study is grounded in several theoretical frameworks that provide a basis for understanding the development of psychological resilience and the potential mechanisms through which mindfulness training may enhance resilience in adolescent athletes.

The transactional theory of stress and coping, proposed by Lazarus and Folkman, posits that an individual’s response to stress is determined by their cognitive appraisal of the stressor and their perceived coping resources. According to this theory, resilience can be conceptualized as a process of successful adaptation to stress, involving the use of effective coping strategies and the ability to maintain positive functioning despite adversity. Mindfulness training may promote resilience by altering athletes’ appraisal processes, encouraging a more accepting and less reactive stance toward stressors, and expanding their repertoire of coping skills.

Cognitive-behavioral theories, such as Beck’s cognitive model and Ellis’ rational-emotive behavior therapy, emphasize the role of cognitions and beliefs in shaping emotional and behavioral responses. These theories suggest that maladaptive thought patterns and irrational beliefs can contribute to psychological distress and hinder resilience. Mindfulness-based interventions, which often incorporate elements of cognitive-behavioral therapy, aim to help individuals become more aware of their thought processes, challenge dysfunctional beliefs, and develop more adaptive and flexible ways of thinking. By promoting cognitive flexibility and reducing cognitive distortions, mindfulness training may foster resilience in the face of stress and adversity.

The broaden-and-build theory of positive emotions, developed by Fredrickson, proposes that positive emotions broaden an individual’s thought-action repertoire and build enduring personal resources, including psychological resilience. According to this theory, the cultivation of positive emotions, such as gratitude, joy, and contentment, can lead to greater resilience over time. Mindfulness practice has been shown to increase positive affect and reduce negative emotions, potentially contributing to the development of resilience through the broaden-and-build process.

Self-determination theory, put forth by Deci and Ryan, emphasizes the importance of autonomy, competence, and relatedness in promoting psychological well-being and optimal functioning. This theory suggests that individuals are more likely to thrive and demonstrate resilience when their basic psychological needs are met. Mindfulness training may support resilience by enhancing athletes’ sense of autonomy (through increased self-awareness and self-regulation), competence (through improved attentional control and coping skills), and relatedness (through greater acceptance and compassion toward oneself and others).

These theoretical frameworks provide a foundation for understanding the complex interplay of factors that contribute to psychological resilience and the potential pathways through which mindfulness training may enhance resilience in adolescent athletes. By integrating these theories, the current study aims to advance our understanding of the mechanisms underlying resilience development and inform the design of effective interventions for this population.

## 3 Research methods

### 3.1 Research participants

This study employed a quantitative research approach using a randomized controlled trial design. The study participants were recruited from a provincial sports school in China. The selection criteria included: (1) aged between 14 and 18 years old; (2) actively engaged in competitive sports training; (3) had at least two years of experience in their respective sports; (4) no history of mental disorders or severe physical injuries; and (5) voluntary participation with informed consent from both the athletes and their legal guardians.

Sample size calculation was conducted based on an expected effect size of *d* = 0.8, power of 0.80, and *α* = 0.05, using the formula: *n* = 2[(Zα/2 + Zβ)^2^σ^2^]/δ^2^. This yielded a minimum requirement of 26 participants per group. Accounting for a potential 20% dropout rate, 30 participants per group were recruited.

A total of 60 adolescent athletes were selected and randomly assigned to either the experimental group (*n* = 30) or the control group (*n* = 30). The experimental group participated in an 8-week mindfulness training program, while the control group maintained their regular training routine without any additional intervention.

The basic information of the research participants is presented in Table 1.

**Table 1:** Basic information of research participants.

Gender	Age (years)	Training experience (years)	Training time (hours/week)	Competition level	Injury history
Male	14–16	2–4	10–15	Provincial	None
Female	14–16	2–4	10–15	Provincial	None
Male	16–18	4–6	15–20	National	Minor
Female	16–18	4–6	15–20	National	Minor

The comparison of the basic characteristics between the experimental group and the control group is shown in Table 2. Independent samples t-tests revealed no significant differences in age (*t* = 0.32, *p* > 0.05) or training experience (*t* = 0.47, *p* > 0.05) between the two groups, indicating that they were comparable at baseline.

**Table 2:** Comparison of basic characteristics between experimental and control groups.

Group	Sample size	Age (years, M ± SD)	Training experience (years, M ± SD)
Experimental	30	15.73 ± 1.26	3.87 ± 1.22
Control	30	15.60 ± 1.35	3.67 ± 1.09
*t*-value		0.32	0.47
*p*-value		>0.05	>0.05

The study was approved by the institutional ethics committee and conducted in accordance with the Declaration of Helsinki. All participants and their legal guardians provided written informed consent prior to the commencement of the study. Confidentiality and anonymity were ensured throughout the research process.

### 3.2 Research instruments

The following measurement tools were employed in this study, selected based on their theoretical relationships and validation in adolescent populations:

1. Connor-Davidson Resilience Scale (CD-RISC): Serving as the primary outcome measure, the CD-RISC is a 25-item self-report scale that assesses an individual’s ability to cope with stress and adversity. Each item is rated on a 5-point Likert scale, with higher scores indicating greater resilience. The scale has demonstrated good internal consistency (Cronbach’s *α* = 0.89) and convergent validity with other measures of resilience and mental health. It has been widely used in research on resilience among various populations, including athletes.
2. Mindful Attention Awareness Scale (MAAS): The MAAS is a 15-item self-report scale that measures an individual’s dispositional mindfulness, or their tendency to be attentive to and aware of present-moment experiences in daily life. Items are rated on a 6-point Likert scale, with higher scores reflecting higher levels of mindfulness. The scale has shown good internal consistency (Cronbach’s *α* = 0.87) and test–retest reliability (*r* = 0.81). It has been validated in both general and athletic populations.
3. Perceived Stress Scale (PSS): The PSS is a 10-item self-report scale that assesses an individual’s perception of stress in their life over the past month. Items are rated on a 5-point Likert scale, with higher scores indicating greater perceived stress. The scale has demonstrated good internal consistency (Cronbach’s α = 0.78–0.91) and convergent validity with other measures of stress and psychological well-being. It has been used in research on stress and coping among athletes.
4. Sport Competition Anxiety Test (SCAT): The SCAT is a 15-item self-report scale that measures an athlete’s tendency to experience anxiety in competitive sports situations. Items are rated on a 3-point scale, with higher scores reflecting higher levels of competitive anxiety. The scale has shown good internal consistency (Cronbach’s α = 0.85) and test–retest reliability (*r* = 0.77). It has been widely used in research on anxiety among athletes.

The psychometric properties of the measurement tools used in this study are summarized in Table 3.

**Table 3:** Reliability and validity of research instruments.

Scale	Number of items	Reliability (Cronbach’s α)	Validity (convergent)	Applicability
CD-RISC	25	0.89	Good	Athletes, General
MAAS	15	0.87	Good	Athletes, General
PSS	10	0.78–0.91	Good	Athletes, General
SCAT	15	0.85	Good	Athletes

The selection of these instruments was based on their theoretical relationships within the mindfulness-resilience framework. The CD-RISC served as the primary outcome measure for psychological resilience. The MAAS was included as a potential mechanism variable to examine mindfulness-related changes. The PSS and SCAT were incorporated as secondary outcome measures to assess stress perception and anxiety levels, providing a comprehensive assessment of psychological well-being.

All scales were administered in their Chinese versions, which have been validated in previous studies with Chinese populations. Participants completed the scales before and after the intervention, as well as at a 3-month follow-up. The scales were presented in a randomized order to control for potential order effects. Instructions were provided to ensure that participants understood how to complete each scale accurately.

### 3.3 Experimental design and procedure

This study employed a randomized controlled trial design with pre-test, post-test, and follow-up assessments. The experimental procedure was conducted as follows:

1. Pre-intervention assessment: All participants completed the battery of questionnaires (CD-RISC, MAAS, PSS, SCAT) to establish baseline levels of resilience, mindfulness, perceived stress, and competitive anxiety.
2. Randomization: Participants were randomly assigned to either the experimental group (mindfulness training) or the control group (regular training) using a computer-generated randomization sequence.
3. Intervention: The experimental group participated in an 8-week mindfulness training program, which consisted of weekly 90-min sessions led by a certified mindfulness instructor. The content of the training program is detailed in Table 4. The control group continued their regular training routine without any additional intervention.
4. Post-intervention assessment: Immediately after the 8-week intervention period, all participants completed the same set of questionnaires as in the pre-intervention assessment.
5. Follow-up assessment: Three months after the intervention, all participants completed the questionnaires again to assess the long-term effects of the mindfulness training.

**Table 4:** Content of the mindfulness training intervention program.

Stage	Training content	Duration	Frequency
Week 1	Introduction to mindfulness, breath awareness	90 min	1/week
Week 2	Body scan, mindful stretching	90 min	1/week
Week 3	Mindful yoga, sitting meditation	90 min	1/week
Week 4	Walking meditation, mindfulness in daily life	90 min	1/week
Week 5	Mindfulness in sports, visualization	90 min	1/week
Week 6	Dealing with difficult emotions, self-compassion	90 min	1/week
Week 7	Mindful communication, interpersonal mindfulness	90 min	1/week
Week 8	Integrating mindfulness into training and life	90 min	1/week

The experimental timeline is presented in Table 5.

**Table 5:** Experimental timeline.

Stage	Time	Main tasks
Pre-intervention	Week 0	Baseline assessment, randomization
Intervention	Weeks 1–8	Mindfulness training (experimental) or regular training (control)
Post-intervention	Week 9	Post-intervention assessment
Follow-up	Week 21	Follow-up assessment
Data analysis	Weeks 22–24	Statistical analysis, interpretation, reporting

Data were collected using paper-and-pencil questionnaires administered in a quiet room at the sports school. Participants were given adequate time to complete the questionnaires and were encouraged to ask for clarification if needed. The instructor was present during data collection to ensure that participants understood the instructions and completed the questionnaires independently.

Data were entered into a spreadsheet and checked for accuracy and completeness. Statistical analyses were performed using SPSS software, with a significance level set at *p* < 0.05. Descriptive statistics, independent samples t-tests, paired samples t-tests, and repeated measures ANOVA were used to analyze the data and test the research hypotheses.

## 4 Results and analysis

### 4.1 Data processing methods

Data collected from the questionnaires were entered into SPSS 26.0 software for statistical analysis. Prior to analysis, the data were screened for missing values, outliers, and normality. Missing data were handled using the expectation–maximization algorithm, which is a maximum likelihood estimation method for imputing missing values. Outliers were identified using box plots and were winsorized to the nearest non-outlier value. Normality was assessed using the Shapiro–Wilk test and visual inspection of histograms and Q-Q plots.

Descriptive statistics, including means, standard deviations, and frequencies, were calculated for all study variables. Independent samples t-tests were used to compare the baseline characteristics of the experimental and control groups. The effectiveness of the mindfulness training intervention was examined using a 2 (group: experimental vs. control) × 3 (time: pre-intervention, post-intervention, follow-up) repeated measures analysis of variance (RM-ANOVA). The assumptions of RM-ANOVA, including sphericity and homogeneity of variance, were assessed using Mauchly’s test and Levene’s test, respectively. If the assumptions were violated, appropriate corrections (e.g., Greenhouse-Geisser) were applied.

*Post hoc* pairwise comparisons with Bonferroni adjustment were conducted to examine significant main effects and interactions. Effect sizes were calculated using partial eta-squared ( $\eta_{p}^{2}$ ) for ANOVA and Cohen’s d for pairwise comparisons. The following formulas were used:

1. Partial eta-squared:

$$\eta_{p}^{2}=\frac{SS_{\text{effect}}}{SS_{\text{effect}}+SS_{\text{error}}}$$

where $SS_{\text{effect}}$ is the sum of squares for the effect and $SS_{\text{error}}$ is the sum of squares for the error.

Cohen’s d:

$$d=\frac{M_{1}-M_{2}}{S_{\text{pooled}}}$$

where $M_{1}$ and $M_{2}$ are the means of the two groups and $S_{\text{pooled}}$ is the pooled standard deviation, calculated as:

$$S_{\text{pooled}}=\sqrt{\frac{(n_{1}-1)S_{1}^{2}+(n_{2}-1)S_{2}^{2}}{n_{1}+n_{2}-2}}$$

where $n_{1}$ and $n_{2}$ are the sample sizes and $S_{1}$ and $S_{2}$ are the standard deviations of the two groups.

Statistical significance was set at *p* < 0.05 for all analyses. Results were reported in accordance with the APA style guidelines.

### 4.2 Associations between mindfulness training and psychological resilience

To examine the associations between mindfulness training intervention and psychological resilience, a 2 (group) × 3 (time) repeated measures ANOVA was conducted on the total score and subscale scores of the CD-RISC. The results revealed a significant main difference of time (*F* (2, 116) = 28.64, *p* < 0.001, ηp^2^ = 0.33) and a significant group × time interaction (*F* (2, 116) = 18.92, *p* < 0.001, ηp^2^ = 0.25), indicating that the changes in resilience over time differed between the experimental and control groups.

*Post hoc* comparisons using the Bonferroni correction showed that the experimental group was associated with significant increases in total resilience scores from pre-intervention (*M* = 62.47, SD = 10.21) to post-intervention (*M* = 75.83, SD = 9.56, *p* < 0.001, *d* = 1.35) and from pre-intervention to follow-up (*M* = 77.90, SD = 9.14, *p* < 0.001, *d* = 1.58). In contrast, the control group showed no significant changes in resilience scores across the three time points (*p* > 0.05). Table 6 presents the means, standard deviations, t-values, and *p*-values for each dimension of resilience before and after the intervention.

**Table 6:** Comparison of resilience scores before and after the intervention.

Dimension	Pre-intervention (M ± SD)	Post-intervention (M ± SD)	*t*-value	*p*-value
Personal competence	23.60 ± 4.82	28.73 ± 4.15	6.87	<0.001
Trust in one’s instincts	16.97 ± 3.64	20.50 ± 3.27	5.92	<0.001
Positive acceptance of change	12.23 ± 2.91	15.03 ± 2.60	5.48	<0.001
Control	9.67 ± 2.45	11.57 ± 2.18	4.76	<0.001

Further analysis of the changes in resilience scores across the three time points revealed a significant increasing trend in the experimental group, while the control group remained relatively stable (see Table 7). The effect sizes (Cohen’s d) for the experimental group were large, ranging from 1.35 to 1.58, indicating a substantial improvement in resilience as a result of the mindfulness training.

**Table 7:** Changes in resilience scores across different time points.

Time point	Experimental group (M ± SD)	Control group (M ± SD)	Effect size (d)
Pre-intervention	62.47 ± 10.21	63.10 ± 9.87	–
Post-intervention	75.83 ± 9.56	64.03 ± 10.12	1.35
Follow-up	77.90 ± 9.14	63.67 ± 10.43	1.58

These findings suggest that the 8-week mindfulness training program was associated with enhanced psychological resilience among adolescent athletes. The intervention group demonstrated significant improvements in all dimensions of resilience, including personal competence, trust in one’s instincts, positive acceptance of change, and control. These improvements were maintained at the 3-month follow-up, suggesting sustained associations between mindfulness training and resilience development.

The results are consistent with previous research that has found mindfulness-based interventions to be associated with resilience development in various populations. The potential mechanisms through which mindfulness relates to resilience may include increased awareness and acceptance of one’s thoughts and emotions, improved emotion regulation, and the development of adaptive coping strategies. By cultivating a non-judgmental and present-focused attitude, mindfulness training may be connected with athletes’ ability to respond to stressors and challenges with greater flexibility and resilience.

In summary, the findings of this study provide empirical support for the associations between mindfulness training and psychological resilience development among adolescent athletes. The significant improvements observed in the experimental group highlight the potential of mindfulness-based interventions as a valuable approach for promoting mental health and well-being in this population.

### 4.3 Analysis of moderating variables

To investigate the potential moderating effects of gender and training experience on the relationship between mindfulness training and psychological resilience, a series of 2 (group) × 2 (gender) and 2 (group) × 2 (training experience) repeated measures ANOVAs were conducted. Training experience was dichotomized into less experienced (≤ 3 years) and more experienced (> 3 years) based on the median split of the sample.

The results revealed no significant main effect of gender (*F* (1, 56) = 1.24, *p* = 0.27, $\eta_{p}^{2}$ = 0.02) or interaction effect between gender and group (*F* (1, 56) = 0.68, *p* = 0.41, $\eta_{p}^{2}$ = 0.01) on resilience scores. Similarly, there was no significant main effect of training experience (*F* (1, 56) = 2.87, *p* = 0.10, $\eta_{p}^{2}$ = 0.05) or interaction effect between training experience and group (*F* (1, 56) = 1.92, *p* = 0.17, $\eta_{p}^{2}$ = 0.03). These findings suggest that the associations between mindfulness training intervention and resilience development were not significantly moderated by the participants’ gender or level of training experience.

Table 8 presents the means, standard deviations, *F*-values, and *p*-values for the moderating variables.

**Table 8:** Analysis of moderating variables’ effects.

Variable	Sample size	Mean	Standard deviation	*F*-value	*p*-value
Gender				1.24	0.27
Male	32	70.84	11.25		
Female	28	73.57	10.92		
Training experience				2.87	0.10
≤ 3 years	34	70.12	11.68		
> 3 years	26	74.73	10.07		

Although no significant moderating effects were found, it is worth noting that the mean resilience scores were slightly higher for female athletes (*M* = 73.57, SD = 10.92) compared to male athletes (*M* = 70.84, SD = 11.25) and for more experienced athletes (*M* = 74.73, SD = 10.07) compared to less experienced athletes (*M* = 70.12, SD = 11.68). These differences, however, did not reach statistical significance.

The lack of significant moderating effects in this study may be attributed to several factors. First, the sample size might not have been large enough to detect small to moderate moderation effects. Second, the dichotomization of training experience based on the median split might have reduced the variability in the data, potentially masking any significant effects. Future research could consider examining training experience as a continuous variable or using alternative cut-off points for categorization.

It is also possible that other factors, such as sport type, competitive level, or individual differences in personality and coping styles, may have a more significant impact on the relationship between mindfulness training and resilience. Further research is needed to explore these potential moderators and their influence on the effectiveness of mindfulness-based interventions for athletes.

In summary, the findings of this study suggest that the mindfulness training intervention was equally effective in promoting psychological resilience among adolescent athletes, regardless of their gender or level of training experience. These results underscore the generalizability of mindfulness-based approaches for enhancing resilience in this population. However, future research with larger sample sizes and more diverse athlete characteristics is warranted to further investigate potential moderators and boundary conditions of the intervention’s effectiveness.

## 5 Conclusion

### 5.1 Summary of research findings

The present study investigated the associations between an 8-week mindfulness training intervention and psychological resilience development among adolescent athletes. The results demonstrated that the intervention group was associated with significant improvements in resilience, as measured by the Connor-Davidson Resilience Scale, compared to the control group. These improvements were observed across all dimensions of resilience, including personal competence, trust in one’s instincts, positive acceptance of change, and control. Moreover, the beneficial effects of the mindfulness training were maintained at the 3-month follow-up, indicating the long-term sustainability of the intervention’s impact.

Further analysis revealed that the effectiveness of the mindfulness training was not significantly moderated by the participants’ gender or level of training experience. This finding suggests that the intervention can be equally beneficial for male and female athletes, as well as for those with varying levels of experience in their respective sports.

### 5.2 Practical implications

The findings of this study have important practical implications for the development and implementation of mental health interventions for adolescent athletes. Given the significant associations between mindfulness training and resilience improvements observed in the intervention group, mindfulness training should be considered a valuable approach for promoting mental well-being and supporting the development of stress management skills in this population.

Sport psychologists, coaches, and athletic organizations can incorporate mindfulness-based practices into their training programs to help young athletes develop the psychological skills necessary for optimal performance and overall well-being. The specific techniques and exercises used in this study, such as breath awareness, body scan, and mindful movement, can be easily adapted and integrated into existing training routines.

Furthermore, the lack of significant moderating effects of gender and training experience highlights the potential for mindfulness interventions to be widely applicable and beneficial for diverse groups of athletes. This underscores the importance of making such interventions accessible to all young athletes, regardless of their background or level of experience.

### 5.3 Limitations and future directions

Despite the promising findings, this study has several limitations that should be acknowledged. First, while the randomized controlled design allows for examination of associations, causal inferences should be made with caution due to the complexity of psychological development processes. Second, the sample size was relatively small, which may have limited the power to detect significant moderating relationships. Future research should aim to replicate these findings with larger and more diverse samples of adolescent athletes.

Second, the study relied solely on self-report measures of resilience, which may be subject to response bias. Future studies could incorporate objective measures, such as physiological indicators of stress or performance outcomes, to provide a more comprehensive assessment of the intervention’s impact.

Third, the long-term effects of the mindfulness training were assessed only at a 3-month follow-up. It would be valuable for future research to examine the sustainability of the intervention’s benefits over longer periods, such as 6 months or 1 year.

Finally, this study focused specifically on the impact of mindfulness training on psychological resilience. Future research could explore the potential effects of mindfulness interventions on other important outcomes for adolescent athletes, such as performance, injury prevention, and overall mental health.

In conclusion, this study provides empirical evidence for the associations between mindfulness training and enhanced psychological resilience among adolescent athletes. The findings highlight the potential of mindfulness-based interventions as a valuable approach for promoting mental well-being and supporting resilience development in this population. Further research is needed to establish causal mechanisms and explore the long-term sustainability of these associations. Further research is needed to replicate these findings, explore potential moderators and mediators, and investigate the long-term sustainability of the intervention’s effects.

## Data Availability

The original contributions presented in the study are included in the article/supplementary material, further inquiries can be directed to the corresponding author.

## References

[ref1] Balcombe L. De Leo D. Turner M. J. (2022). Editorial: athlete psychological resilience and digital mental health implementation. Front. Psychol. 13:1082998. doi: 10.3389/fpsyg.2022.1082998, PMID: 36532980 PMC9757057

[ref2] Baltzell A. (2016). Mindfulness and performance. Cambridge, UK: Cambridge University Press.

[ref3] Bernier M. Thienot E. Codron R. Fournier J. F. (2009). Mindfulness and acceptance approaches in sport performance. J. Clin. Sport Psychol. 3, 320–333. doi: 10.1123/jcsp.3.4.320

[ref4] Birrer D. Röthlin P. Morgan G. (2012). Mindfulness to enhance athletic performance: theoretical considerations and possible impact mechanisms. Mindfulness 3, 235–246. doi: 10.1007/s12671-012-0109-2

[ref5] Brenner J. S. (2024). Mindfulness for young athletes. Sports Health. 16, 300–302. doi: 10.1177/19417381231209219, PMID: 37936388 PMC10916786

[ref6] Brown K. W. Ryan R. M. (2003). The benefits of being present: mindfulness and its role in psychological well-being. J. Pers. Soc. Psychol. 84, 822–848. doi: 10.1037/0022-3514.84.4.822, PMID: 12703651

[ref7] Bühlmayer L. Birrer D. Röthlin P. Faude O. Donath L. (2017). Effects of mindfulness practice on performance-relevant parameters and performance outcomes in sports: a meta-analytical review. Sports Med. 47, 2309–2321. doi: 10.1007/s40279-017-0752-9, PMID: 28664327

[ref8] Chen S. Yuan J. Yao J. Jin Y. Xie X. Wei D. (2024). The effect of mindfulness training on the psychological state of high-level athletes: meta analysis and system evaluation research. J. Sports Sci. 43, 600–622. doi: 10.1080/02640414.2025.2468997 40010739

[ref9] Collins D. MacNamara Á. (2012). The rocky road to the top: why talent needs trauma. Sports Med. 42, 907–914. doi: 10.1007/BF03262302, PMID: 23013519

[ref10] Connor K. M. Davidson J. R. T. (2003). Development of a new resilience scale: the Connor-Davidson resilience scale (CD-RISC). Depress. Anxiety 18, 76–82. doi: 10.1002/da.10113 12964174

[ref11] Den Hartigh R. J. R. Gernigon C. Van Yperen N. W. Marin L. Van Geert P. L. C. (2022). Resilience in sports: a multidisciplinary, dynamic, and personalized perspective. Int. Rev. Sport Exerc. Psychol. 17, 564–586. doi: 10.1080/1750984X.2022.2039749, PMID: 38835409 PMC11147456

[ref12] Fletcher D. Sarkar M. (2012). A grounded theory of psychological resilience in Olympic champions. Psychol. Sport Exerc. 13, 669–678. doi: 10.1016/j.psychsport.2012.04.007

[ref13] Fletcher D. Sarkar M. (2013). Psychological resilience: a review and critique of definitions, concepts, and theory. Eur. Psychol. 18, 12–23. doi: 10.1027/1016-9040/a000124

[ref14] Fredrickson B. L. (2004). The broaden-and-build theory of positive emotions. Philos. Trans. R. Soc. B 359, 1367–1377. doi: 10.1098/rstb.2004.1512, PMID: 15347528 PMC1693418

[ref15] Galli N. Gonzalez S. P. (2015). Psychological resilience in sport: a review of the literature and implications for research and practice. Int. J. Sport Exerc. Psychol. 13, 243–257. doi: 10.1080/1612197X.2014.946947

[ref16] Galli N. Vealey R. S. (2008). "bouncing back" from adversity: athletes' experiences of resilience. Sport Psychol. 22, 316–335. doi: 10.1123/tsp.22.3.316

[ref17] Gardner F. L. Moore Z. E. (2007). The psychology of enhancing human performance: The mindfulness-acceptance-commitment (MAC) approach. New York, NY: Springer Publishing Company.

[ref18] Gonzalez S. P. Moore E. W. G. Newton M. Galli N. A. (2016). Validity and reliability of the Connor-Davidson resilience scale (CD-RISC) in competitive sport. Psychol. Sport Exerc. 23, 31–39. doi: 10.1016/j.psychsport.2015.10.005

[ref19] Gucciardi D. F. Jackson B. Coulter T. J. Mallett C. J. (2011). The Connor-Davidson resilience scale (CD-RISC): dimensionality and age-related measurement invariance with Australian cricketers. Psychol. Sport Exerc. 12, 423–433. doi: 10.1016/j.psychsport.2011.02.005

[ref20] Gupta S. McCarthy P. J. (2022). The sporting resilience model: a systematic review of resilience in sport performers. Front. Psychol. 13:1003053. doi: 10.3389/fpsyg.2022.1003053, PMID: 36619099 PMC9811683

[ref21] Hardy L. Arthur C. A. Jones G. Shariff A. Munnoch K. Isaacs I. . (2010). The relationship between transformational leadership behaviors, psychological, and training outcomes in elite military recruits. Leadersh. Q. 21, 20–32. doi: 10.1016/j.leaqua.2009.10.002

[ref22] Harman B. Dessart G. Puke L. Philippe R. A. (2022). Coping and resilience among endurance athletes during the COVID-19 pandemic. Front. Psychol. 13:811499. doi: 10.3389/fpsyg.2022.811499, PMID: 35664192 PMC9161140

[ref23] Hill Y. Den Hartigh R. J. R. Meijer R. R. De Jonge P. Van Yperen N. W. (2018). Resilience in sports from a dynamical perspective. Sport Exerc. Perform. Psychol. 7, 333–341. doi: 10.1037/spy0000118

[ref24] Hosseini S. A. Besharat M. A. (2010). Relation of resilience with sport achievement and mental health in a sample of athletes. Procedia Soc. Behav. Sci. 5, 633–638. doi: 10.1016/j.sbspro.2010.07.156

[ref25] Hussain T. Wang D. Li B. (2023). Psychological resilience in athletes during the COVID-19 pandemic: a qualitative insight. Acta Psychol. 240:104050. doi: 10.1016/j.actpsy.2023.104050, PMID: 37832493

[ref26] Josefsson T. Gustafsson H. Robinson P. Cedenblad M. Sievert E. Ivarsson A. (2024). Examining the effects of the mindfulness-acceptance-commitment (MAC) programme on sport-specific dispositional mindfulness, sport anxiety, and experiential acceptance in martial arts. Scand. J. Sport Exerc. Psychol. 6, 19–26. doi: 10.7146/sjsep.v6i.140996

[ref27] Josefsson T. Ivarsson A. Gustafsson H. Stenling A. Lindwall M. Tornberg R. . (2017). Effects of mindfulness-acceptance-commitment (MAC) on sport-specific dispositional mindfulness, emotion regulation, and self-rated athletic performance in a multiple-sport population: an RCT study. Mindfulness 10, 669–678. doi: 10.1007/s12671-019-01098-7

[ref28] Kalkhoven J. T. Watsford M. L. Impellizzeri F. M. (2020). A conceptual model and detailed framework for stress-related, strain-related, and overuse athletic injury. J. Sci. Med. Sport 23, 726–734. doi: 10.1016/j.jsams.2020.02.002, PMID: 32111566

[ref29] Kaufman K. A. Glass C. R. Arnkoff D. B. (2009). Evaluation of mindful sport performance enhancement (MSPE): a new approach to promote flow in athletes. J. Clin. Sport Psychol. 3, 334–356. doi: 10.1123/jcsp.3.4.334

[ref30] Kaufman K. A. Glass C. R. Pineau T. R. (2018). Mindful sport performance enhancement: Mental training for athletes and coaches. Washington, DC: American Psychological Association.

[ref31] Lazarus R. S. Folkman S. (1984). Stress, appraisal, and coping. New York, NY: Springer.

[ref32] Li H. Liu M. Long T. Chen W. Tian Y. (2023). A longitudinal and multilevel investigation of grateful climate in cultivating psychological resilience: the mediating role of athlete's gratitude. Appl. Res. Qual. Life 18, 2985–3004. doi: 10.1007/s11482-023-10259-9

[ref33] Myall K. Montero-Marin J. Gorczynski P. Kajee N. Sheriff R. S. Bernard R. . (2023). Effect of mindfulness-based programmes on elite athlete mental health: a systematic review and meta-analysis. Br. J. Sports Med. 57, 99–108. doi: 10.1136/bjsports-2022-105596, PMID: 36223914

[ref34] Noetel M. Ciarrochi J. Van Zanden B. Lonsdale C. (2019). Mindfulness and acceptance approaches to sporting performance enhancement: a systematic review. Int. Rev. Sport Exerc. Psychol. 12, 139–175. doi: 10.1080/1750984X.2017.1387803

[ref35] Nuetzel B. Ebert A. Pittig A. Tuschen-Caffier B. (2023). Coping strategies for handling stress and providing mental health in elite athletes: a systematic review. Front. Sports Act. Living 5:1265783. doi: 10.3389/fspor.2023.1265783 38033656 PMC10687549

[ref36] Reardon C. L. Hainline B. Aron C. M. Baron D. Baum A. L. Bindra A. . (2019). Mental health in elite athletes: International Olympic Committee consensus statement (2019). Br. J. Sports Med. 53, 667–699. doi: 10.1136/bjsports-2019-100715, PMID: 31097450

[ref37] Roth L. H. O. Bencker C. Lorenz J. Laireiter A.-R. (2024). Testing the validity of the broaden-and build theory of positive emotions: a network analytic approach. Front. Psychol. 15:1405272. doi: 10.3389/fpsyg.2024.1405272, PMID: 39380763 PMC11458511

[ref38] Rutter M. (2006). “The promotion of resilience in the face of adversity” in Families count: Effects on child and adolescent development. eds. Clarke-Stewart A. Dunn J. (Cambridge, UK: Cambridge University Press), 26–52.

[ref39] Ryan R. M. Deci E. L. (2020). Intrinsic and extrinsic motivation from a self-determination theory perspective: definitions, theory, practices, and future directions. Educ. Psychol. 55, 166–176. doi: 10.1080/00461520.2020.1724062

[ref40] Sarkar M. Fletcher D. (2014). Psychological resilience in sport performers: a review of stressors and protective factors. J. Sports Sci. 32, 1419–1434. doi: 10.1080/02640414.2014.901551, PMID: 24716648

[ref41] Seery M. D. (2011). Resilience: a silver lining to experiencing adverse life events? Curr. Dir. Psychol. Sci. 20, 390–394. doi: 10.1177/0963721411424740

[ref42] Smith R. E. Schutz R. W. Smoll F. L. Ptacek J. T. (1995). Development and validation of a multidimensional measure of sport-specific psychological skills: the athletic coping skills Inventory-28. J. Sport Exerc. Psychol. 17, 379–398. doi: 10.1123/jsep.17.4.379

[ref43] Su N. Si G. Liang W. Bu D. Jiang X. (2024). Mindfulness and acceptance-based training for elite adolescent athletes: a mixed-method exploratory study. Front. Psychol. 15:1401763. doi: 10.3389/fpsyg.2024.1401763, PMID: 38860040 PMC11163101

[ref44] Wagstaff C. R. D. Hings R. F. Larner R. Fletcher D. (2018). Psychological resilience's moderation of the relationship between the frequency of organizational stressors and burnout in athletes and coaches. The Sport Psychologist 32, 178–188. doi: 10.1123/tsp.2016-0068

[ref45] Wang Y. Lei S. M. Fan J. (2023). Effects of mindfulness-based interventions on promoting athletic performance and related factors among athletes: a systematic review and meta-analysis of randomized controlled trial. Int. J. Environ. Res. Public Health 20:2038. doi: 10.3390/ijerph20032038, PMID: 36767403 PMC9915077

